# Supplementary figures and images for: Regulation of canonical Wnt signalling by the ciliopathy protein MKS1 and the E2 ubiquitin-conjugating enzyme UBE2E1
Source: eLife. 2022 Feb 16;11:e57593. doi: 10.7554/eLife.57593 (PMC8880992; doi:10.7554/eLife.57593)

Figure 1a P-b-cat and b-catenin

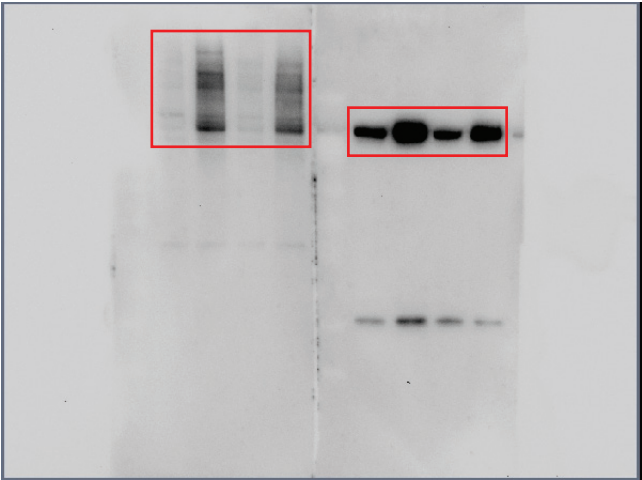

Figure 1a b-actin

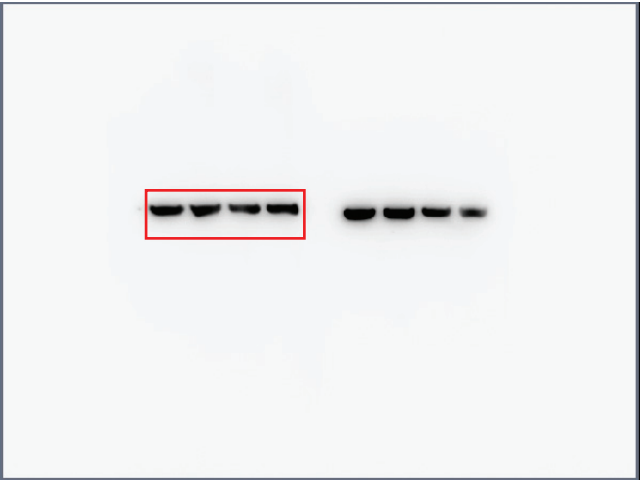

Figure 1c 20Sa7

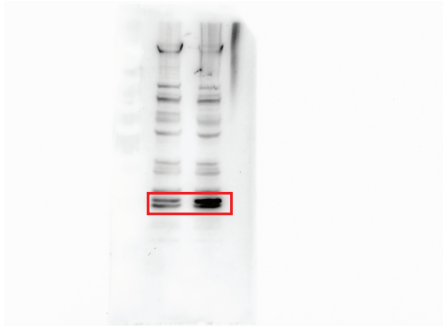

Figure 1c b-actin

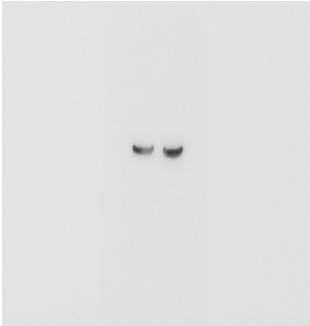

Supplement: Figure 1—source data 1. [file elife-57593-fig1-data1.pdf]

Figure 1 fs 1a

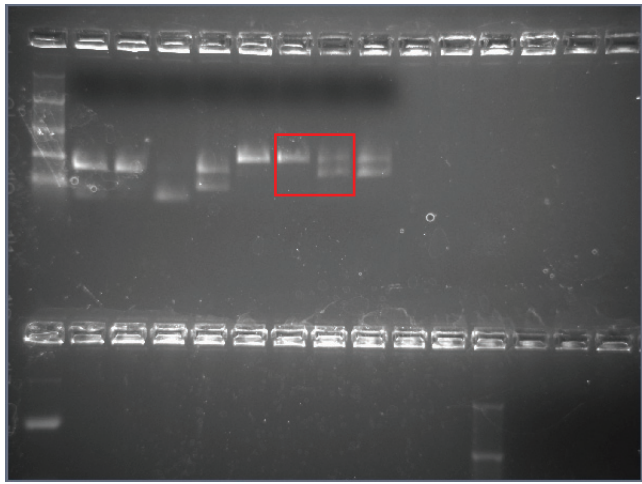

Figure 1 fs 1a

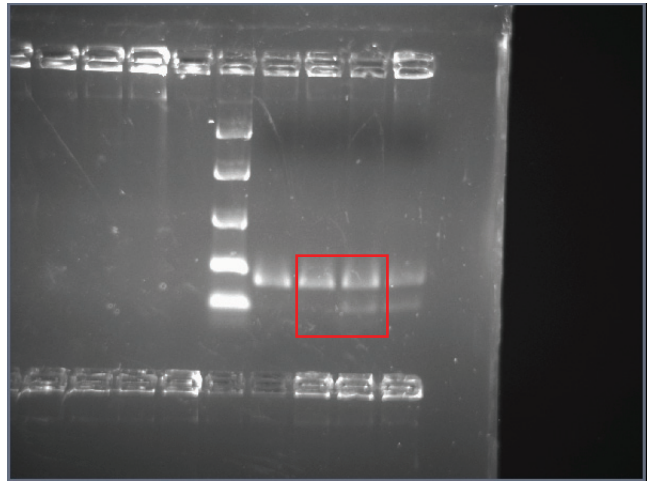

Figure 1 fs 1b MKS1

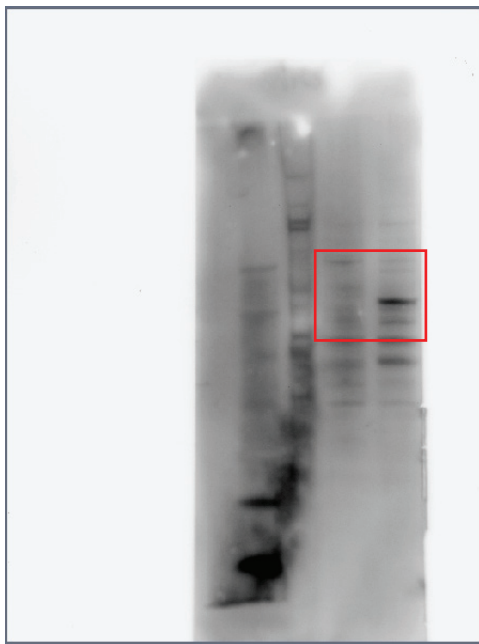

Figure 1 fs 1b b-actin

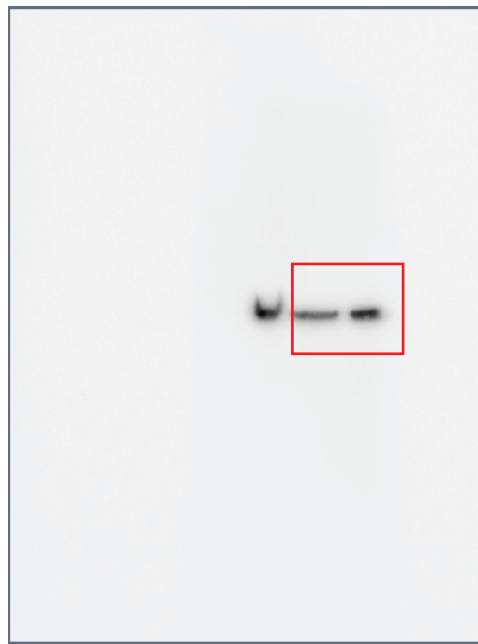

Supplement: Figure 1—figure supplement 1—source data 1. [file elife-57593-fig1-figsupp1-data1.pdf]

Figure 2d GST

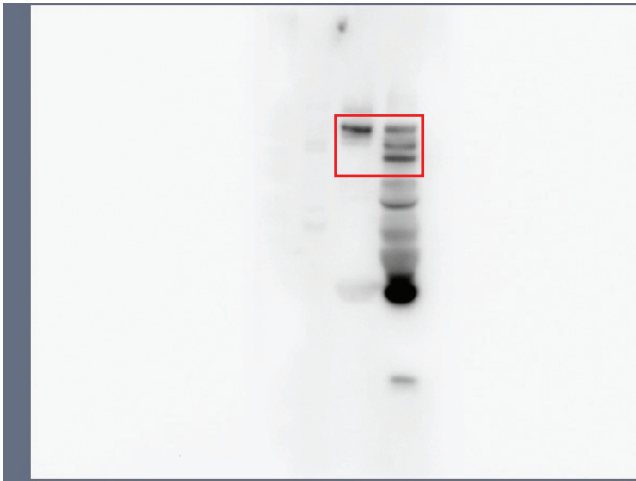

Figure 2d MKS1

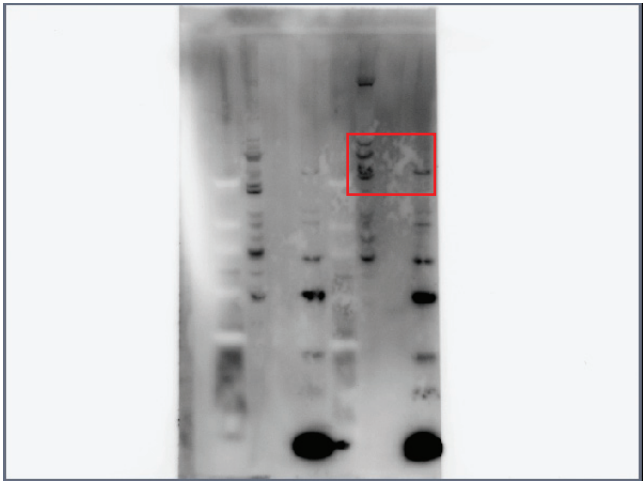

Figure 2e

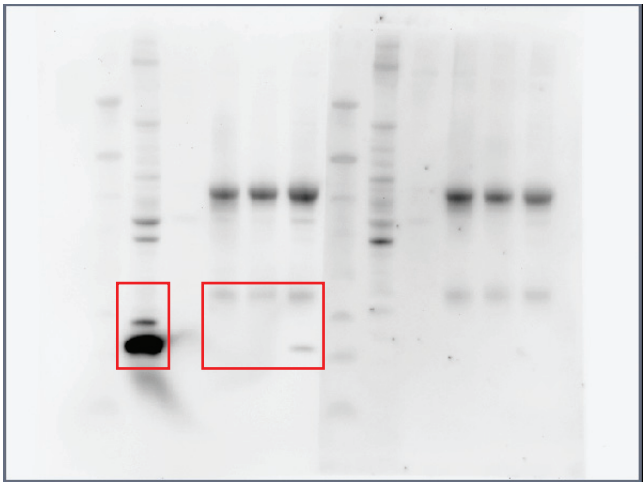

Figure 2f

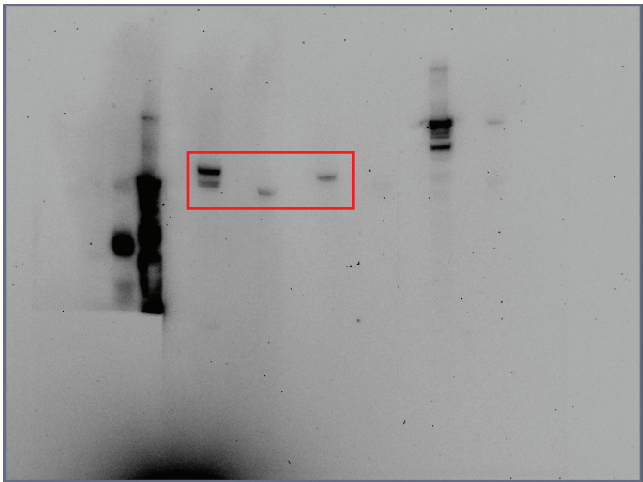

Supplement: Figure 2—source data 1. [file elife-57593-fig2-data1.pdf]

Figure 2 fs 1a

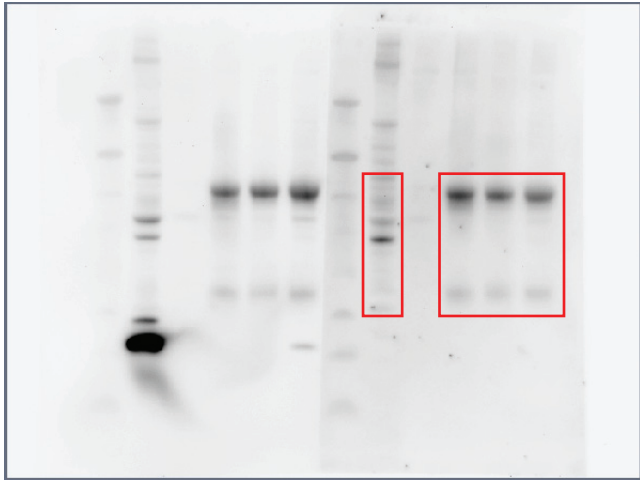

Supplement: Figure 2—figure supplement 1—source data 1. [file elife-57593-fig2-figsupp1-data1.pdf]

Figure 4c

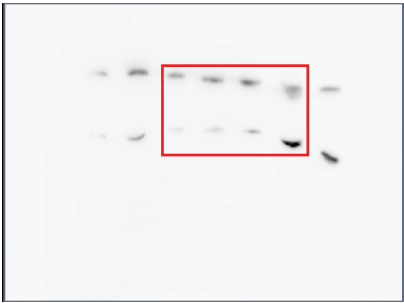

Supplement: Figure 4—source data 1. — The data-set lists proteins (identified by ≥3 unique peptide counts) pulled-down by anti-MKS1 immunoprecipitations, under different conditions of ciliogenesis (proliferating cells, ciliated cells, cells undergoing ciliary resorption). Columns A and B: protein and gene name. Column C: protein accession number. Columns G to L: peptide counts identified by LC-MS/MS mass spectrometry (columns F and M indicating non-specific peptide counts following BSA washes), with counts heat-mapped red (high) to green (low). Column N: χ2 tests of peptide counts for shScr compared to shUbe2e1 knockdown cells, across different conditions of ciliogenesis (red highlighted cells indicate χ2 test p < 0.05). Columns Q to S: shScr:shUbe2e1 peptide count ratios (derived from columns G to L), with values < 1 indicating decreased peptide counts following shUbe2e1 knockdown. Columns U to Z: indicate if a particular protein was identified in a significantly enriched biological process under the indicated GO terms (row 2). [file elife-57593-fig4-data1.pdf]

5c bbeta actin and UBE2E1

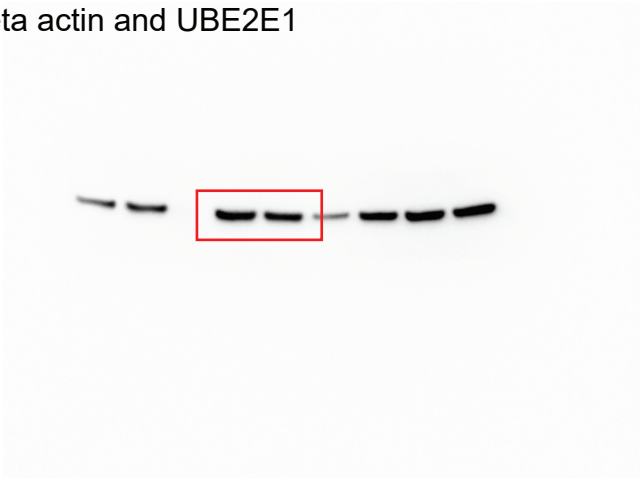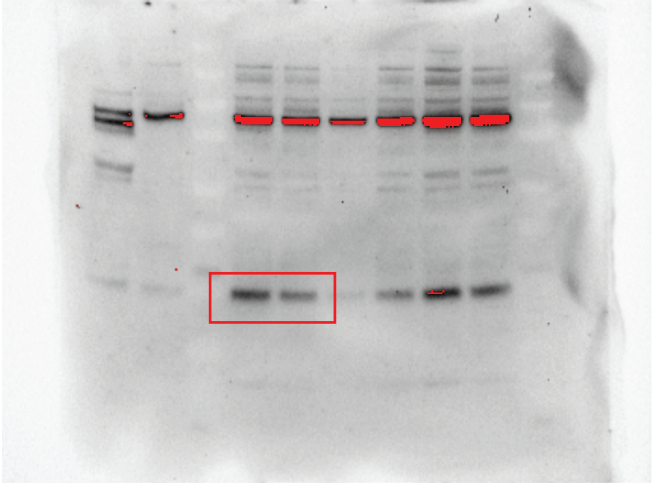

5c beta actin and MKS1

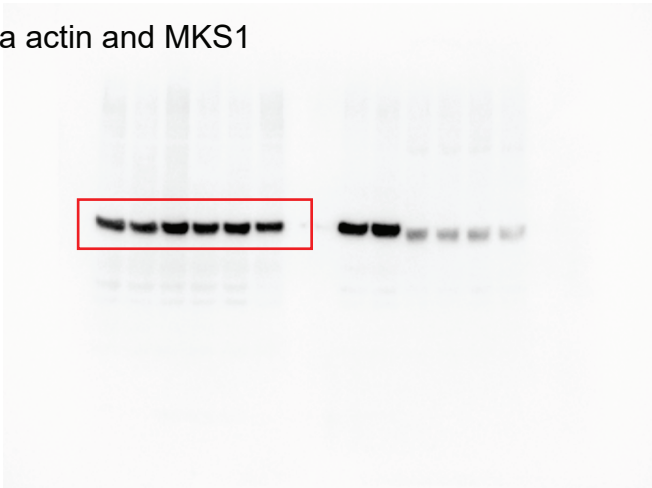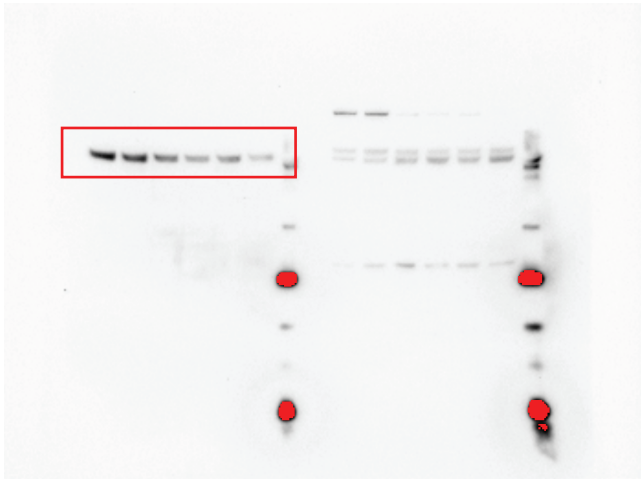

5d cmyc

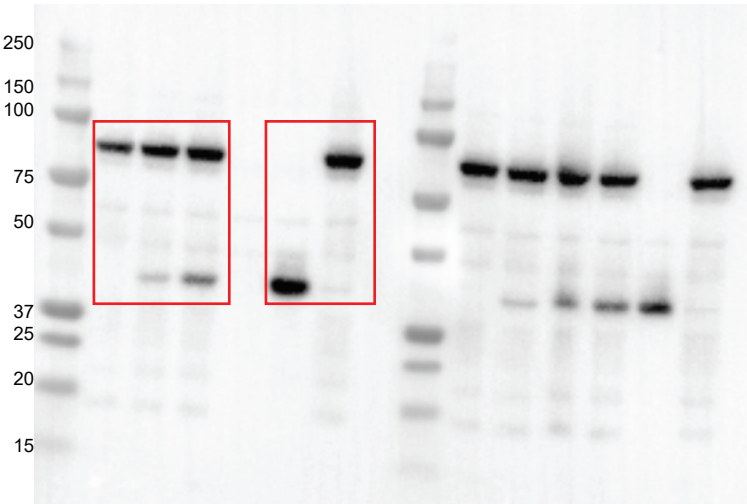

5d beta-actin

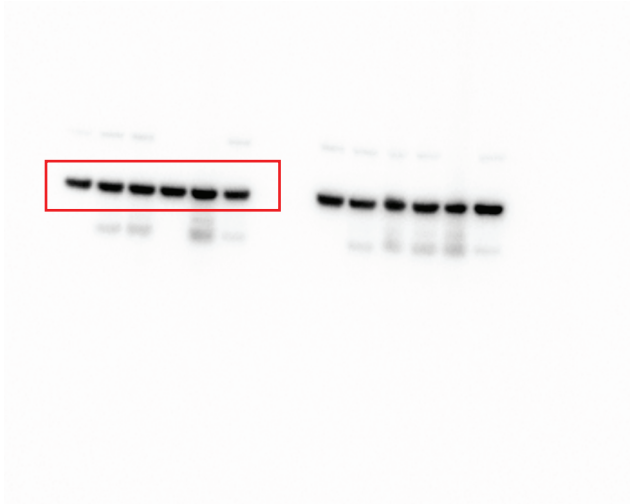

Supplement: Figure 5—source data 1. [file elife-57593-fig5-data1.pdf]

Figure 6a

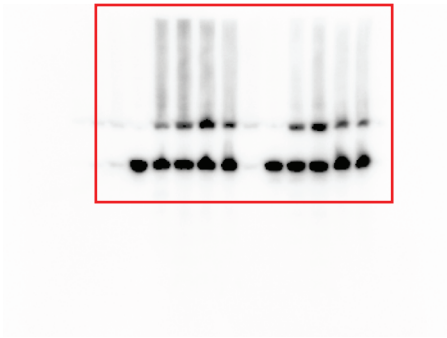

Figure 6b

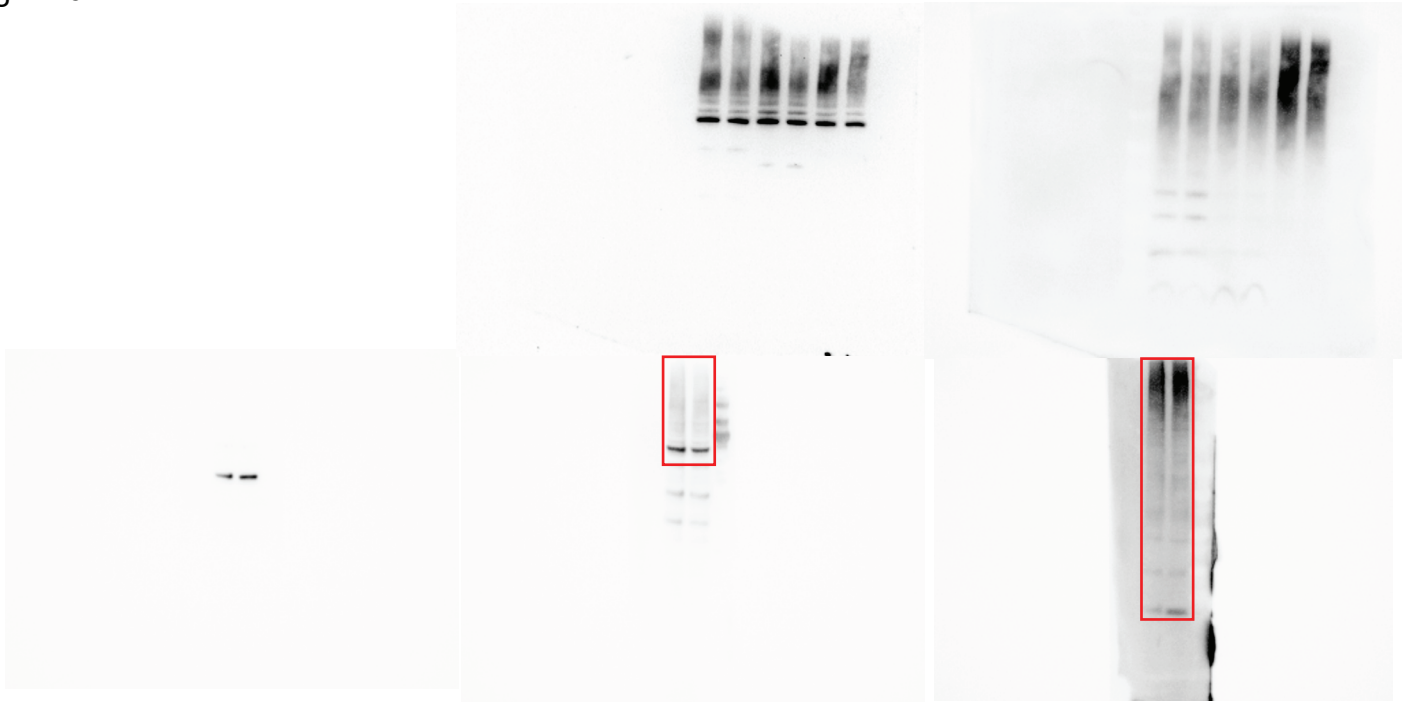

Figure 6c

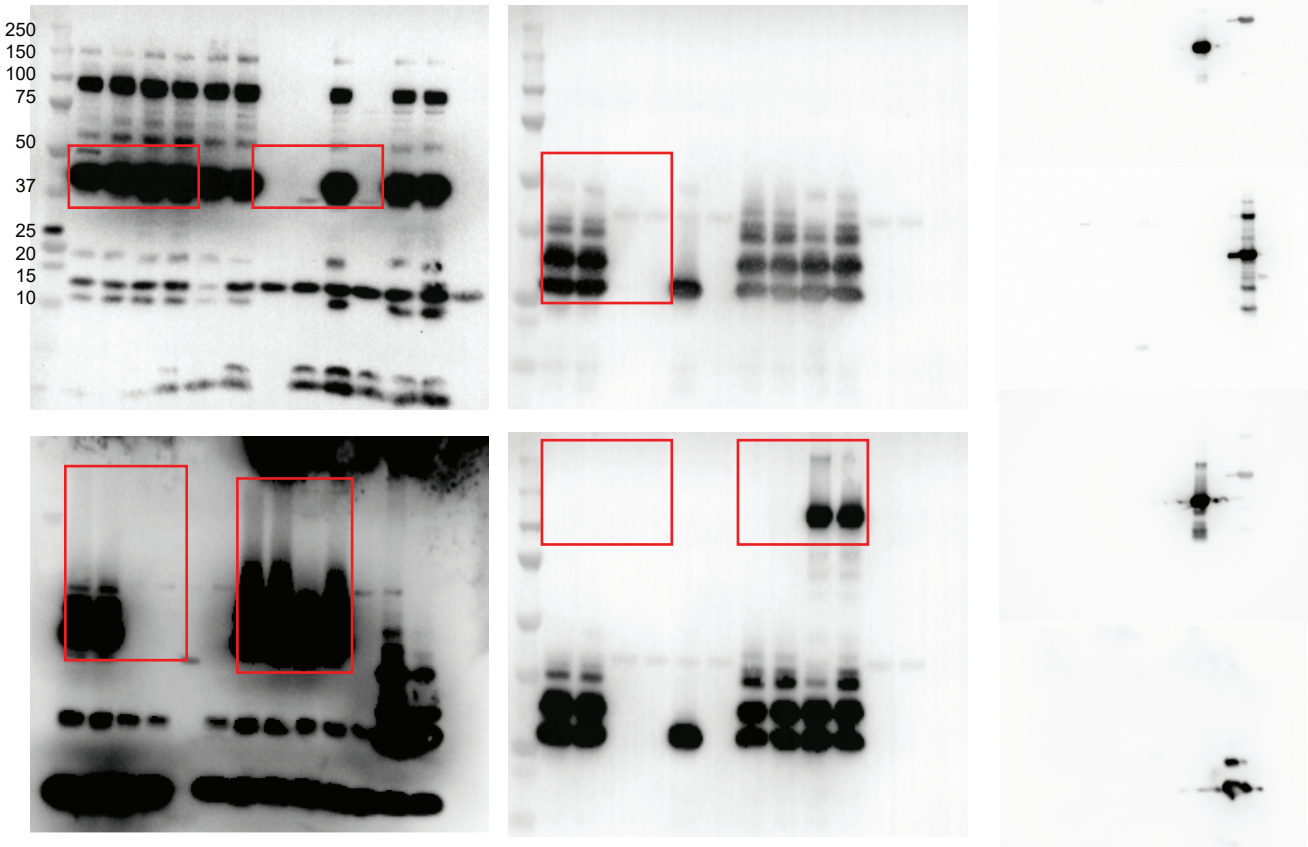

Supplement: Figure 6—source data 1. [file elife-57593-fig6-data1.pdf]

Figure 7a

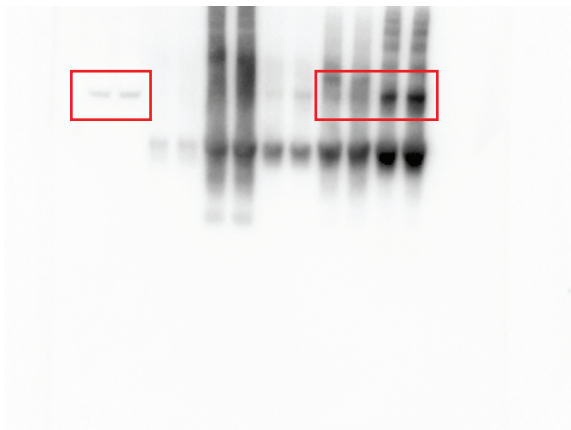

Figure 7b

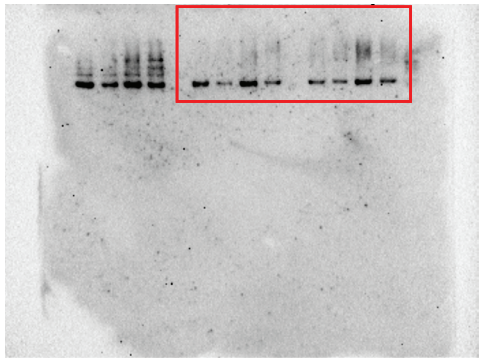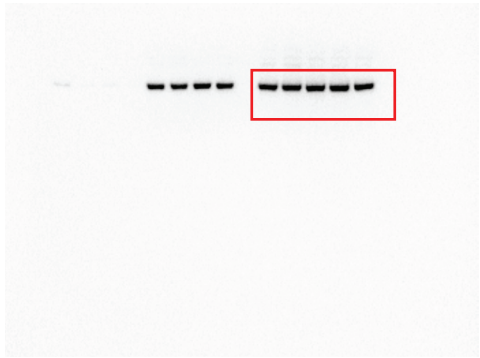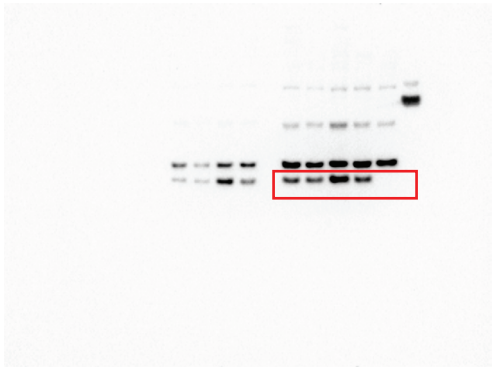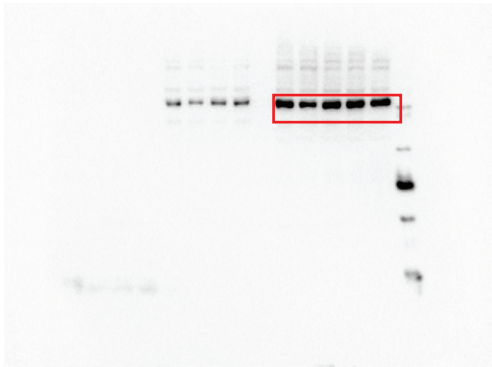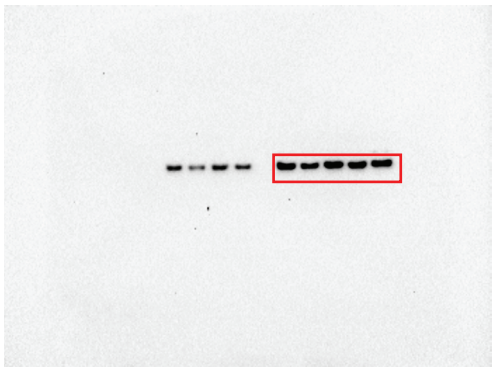

Figure 7c

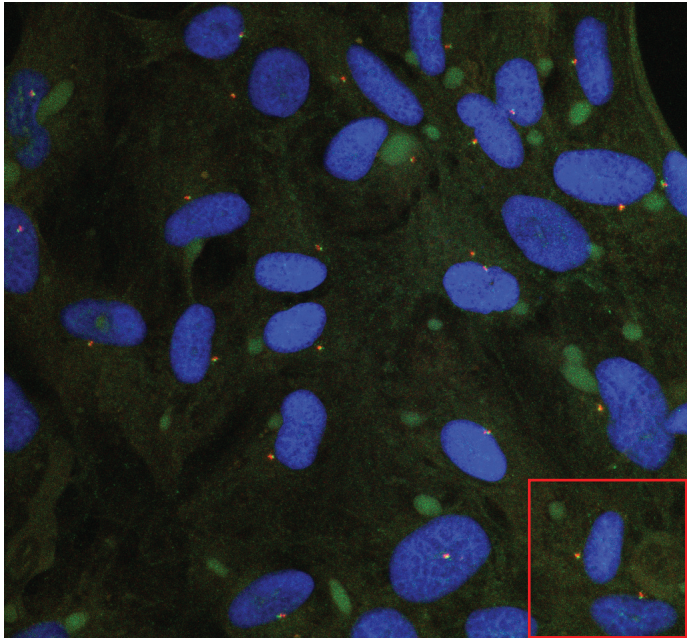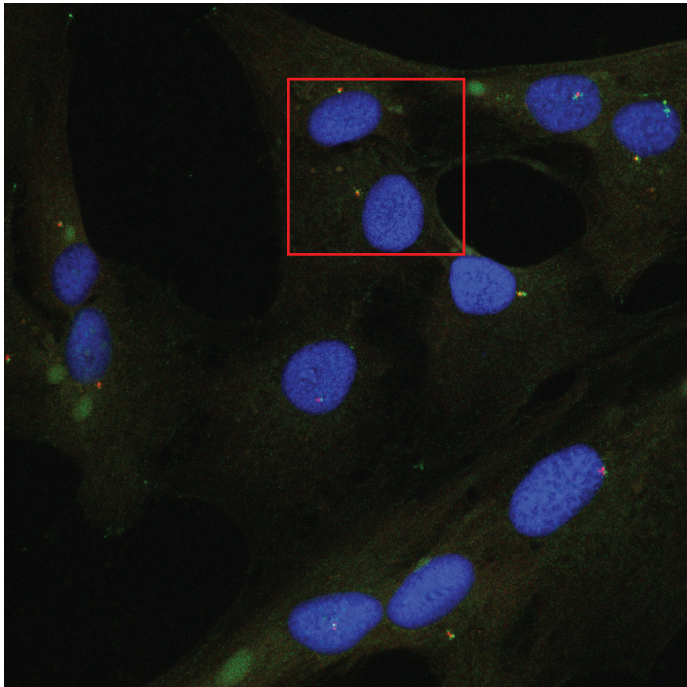

Supplement: Figure 7—source data 1. [file elife-57593-fig7-data1.pdf]
